# The Bacterial Carbon-Fixing Organelle Is Formed by Shell Envelopment of Preassembled Cargo

**DOI:** 10.1371/journal.pone.0076127

**Published:** 2013-09-04

**Authors:** Anna H. Chen, Avi Robinson-Mosher, David F. Savage, Pamela A. Silver, Jessica K. Polka

**Affiliations:** 1 Department of Systems Biology, Harvard Medical School, Boston, Massachusetts, United States of America; 2 Wyss Institute for Biologically Inspired Engineering, Harvard University, Boston, Massachusetts, United States of America; 3 Department of Molecular and Cell Biology, Department of Chemistry and Energy Biosciences Institute, University of California, Berkeley, California, United States of America; HHMI, Massachusetts Institute of Technology, United States of America

## Abstract

**Background:**

Cyanobacteria play a significant role in the global carbon cycle. In *Synechococcus* *elongatus* , the carbon-fixing enzyme ribulose-1,5-bisphosphate carboxylase/oxygenase (RuBisCO) is concentrated into polyhedral, proteinaceous compartments called carboxysomes.

**Methodology/Principal Findings:**

Using live cell fluorescence microscopy, we show that carboxysomes are first detected as small seeds of RuBisCO that colocalize with existing carboxysomes. These seeds contain little or no shell protein, but increase in RuBisCO content over several hours, during which time they are exposed to the solvent. The maturing seed is then enclosed by shell proteins, a rapid process that seals RuBisCO from the cytosol to establish a distinct, solvent-protected microenvironment that is oxidizing relative to the cytosol. These closure events can be spatially and temporally coincident with the appearance of a nascent daughter RuBisCO seed.

**Conclusions/Significance:**

Carboxysomes assemble in a stepwise fashion, inside-to-outside, revealing that cargo is the principle organizer of this compartment’s biogenesis. Our observations of the spatial relationship of seeds to previously formed carboxysomes lead us to propose a model for carboxysome replication via sequential fission, polymerization, and encapsulation of their internal cargo.

## Introduction

Intracellular compartmentalization has long been considered the exclusive province of eukaryotes. However, prokaryotic cells also contain intracellular organelles, falling broadly into two categories. Some compartments are membrane-bound, including *Gemmata* nucleoids [1], cyanobacterial thylakoids [2], and magnetosomes [3]. Others are completely proteinaceous, such as gas vesicles [4] and metabolically active structures termed bacterial microcompartments. These form icosahedral structures that enclose enzymes required for certain metabolic processes, such as ethanolamine and propanediol utilization [5,6].

The carboxysome is one such microcompartment that encapsulates the carbon-fixing enzyme ribulose-1,5-bisphosphate carboxylase (RuBisCO) and carbonic anhydrase [7]. Carboxysomes are found in diverse cyanobacteria and chemoautotrophs and are crucial to the carbon sequestering capabilities of these organisms [8,9]. Inside the carboxysome, carbonic anhydrase converts bicarbonate to CO_2_, which, along with ribulose-1,5-bisphosphate, is consumed by RuBisCO to produce 3-phosphoglycerate. Thus, the carboxysome serves to concentrate the metabolically inefficient RuBisCO enzyme and to increase the local concentration of CO_2_. It has also been proposed that the carboxysome shell is selectively permeable to bicarbonate and ribulose-1,5-bisphophate while excluding oxygen, a competitor substrate of RuBisCO (Kinney et al. 2012). Finally, it has been speculated that the mature carboxysome must maintain a distinct internal oxidative microenvironment to enable the enzymatic activity of carbonic anhydrase [10].

The mechanism and temporal sequence of carboxysome assembly is not known. The interior of the carboxysome is densely packed with its major cargo RuBisCO and a lower concentration of carbonic anhydrase. These are enclosed by proteins that form an icosahedral shell ~100nm in diameter [11,12]. Though their ultrastructural, but not phylogenetic, similarity to viral capsids may suggest that carboxyosomes assemble *de novo*, the mechanism of their biogenesis remains an unsolved problem [5].

There is evidence to suggest that shell proteins and cargo assemble together. Partially assembled carboxysomes have been observed by electron cryotomography, always containing both RuBisCO and shell proteins [13]. However, these data also argue that the cargo must have some intrinsic ability to self-assemble, as RuBisCO is seen to fill the inner layers of the nascent compartment. Indeed, *in vitro* evidence suggests that carboxysome contents can self-associate to form a structure without shell proteins [14]. Shell proteins of some carboxysomes can also independently assemble, forming empty microcompartments in the absence of cargo proteins [15].

The sequence by which these proteins assemble to form this complex organelle is not understood. We employ live cell fluorescence microscopy of *Synechcoccus* *elongatus* PCC 7942 to monitor the dynamics of carboxysome assembly. We find that carboxysomes originate near, and in some cases using material from, preexisting carboxysomes. They are born as small foci of RubisCO, which then grow over a period of hours. Shell proteins colocalize to these foci hours later, abruptly assembling to enclose the compartment and establish a protected internal microenvironment.

## Results

### Growing cells typically assemble one carboxysome at a time

**Figure 1:**
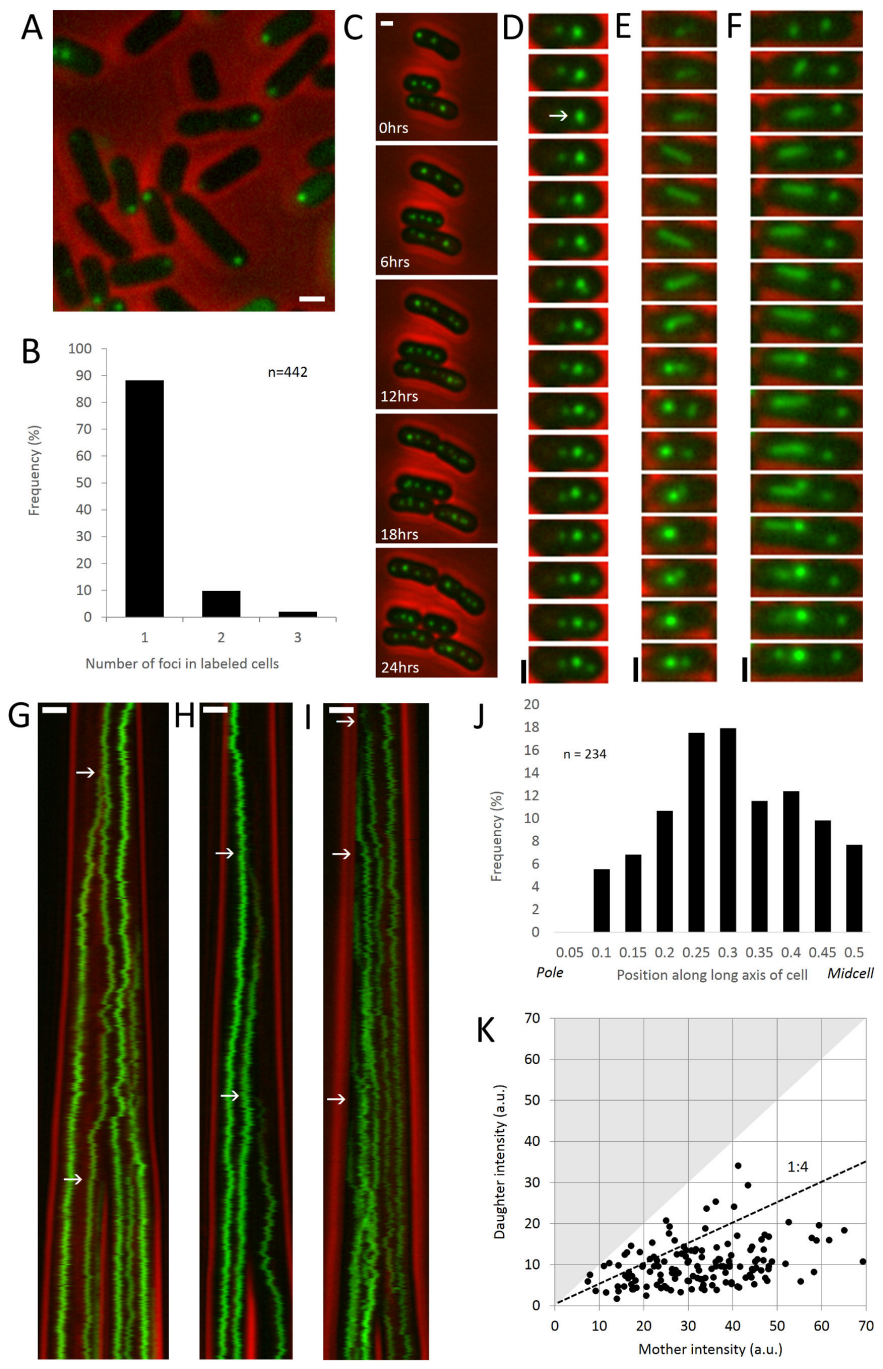
Carboxysomes are born one at a time at the site of preexisting carboxysomes. (A) In pulse-chase labeling of RbcL-SNAP in live *S* *. elongatus* cells, actively assembling carboxysomes with solvent accessible RbcL-SNAP are labeled with BG dye. Red: phase contrast. Green: RbcL. Scale bar: 1µm. (B) The distribution of the number of SNAP labeled carboxysomes, indicating active assembly, in cells directly after labeling (n=442). (C) The biogenesis of carboxysomes can be monitored from long timelapses. Red: phase contrast. Green: RbcL-GFP. Scale bar: 1µm. (D) Montage showing the formation of new carboxysome at the site of a preexisting carboxysome. White arrow indicates the birth event. Panel height: 25 pixels. Time interval: 3 minutes. (E–F) RuBisCO foci elongate into bar carboxysomes that subsequently split into two carboxysomes. Scale bar: 1µm. Time interval: 75 minutes. (G–I) Kymographs of RbcL-GFP in growing and dividing cells. Carboxysome birth events are indicated by white arrows. Scale bar: 1µm. Time interval: 3 minutes. (J) Spatial distribution of 234 birth events along the long axis of the cell. Quarter cell positions are favored. (K) Relative intensity of 141 pairs of new (daughter) carboxysomes and the preexisting carboxysomes to which they initially colocalize (mothers) reveals that birth events are highly asymmetric, with mean daughter intensity being 1/4 that of the mother. Because pairs are sorted into dim (daughter) and bright (mother) pairs, no data points can fall into the shaded area. Dotted line indicates a 1:4 ratio.

Given existing structural evidence, we reasoned that solvent-accessible labeling strategies could be used to mark only RuBisCO inside carboxysomes in the process of forming. We constructed a strain of *S* *. elongatus* with SNAP labeled RuBisCO (RbcL-SNAP) expressed under an IPTG-inducible promoter (Figure 1A). After a 24 hour induction, we pulsed the cells with a fluorescent cell-permeable BG dye for 30 minutes and visualized them with fluorescence microscopy (Figure 1A-B). Among cells containing labeled foci, 88% had one, 10% had two, and 2% had three foci (Figure 1B, n=442). Thus, only a small subset of the average 3.7 carboxysomes per cell are labeled [16]. We used time-lapse microscopy to determine the difference between cells that contained labeled foci and those that did not. We found that all growing and dividing cells contained labeled foci (n=223), and those without labeled foci did not grow or divide. Thus, BG-labeled foci represent carboxysomes being assembling during the labeling pulse, mature carboxysomes are impermeable to the BG dye, and quiescent cells are not generating new carboxysomes. The distribution of foci numbers in growing cells indicates that *S* *. elongatus* carboxysomes are assembled one at a time rather than in parallel, as has been observed by electron microscopy in other genera of cyanobacteria [13].

### New carboxysome are born colocalized with preexisting carboxysomes

Using time-lapse microscopy, we visualized the biogenesis of carboxysomes in live *S* *. elongatus* cells with green fluorescent protein labeled RuBisCO (RbcL-GFP) expressed under the IPTG-inducible promoter (Figure 1C). Fusions at this locus produce an additional 11% of wild-type levels of RbcL (Figure S1) and do not restrict growth [16]. We observed that new carboxysomes are formed at the site of preexisting carboxysomes. At the beginning of each birth event, a preexisting focus of RuBisCO is sometimes seen to take on an asymmetric character (Figure 1D, white arrow). Subsequently, a dimmer daughter focus emerges from the brighter mother carboxysome (Video S1).

**Figure 2:**
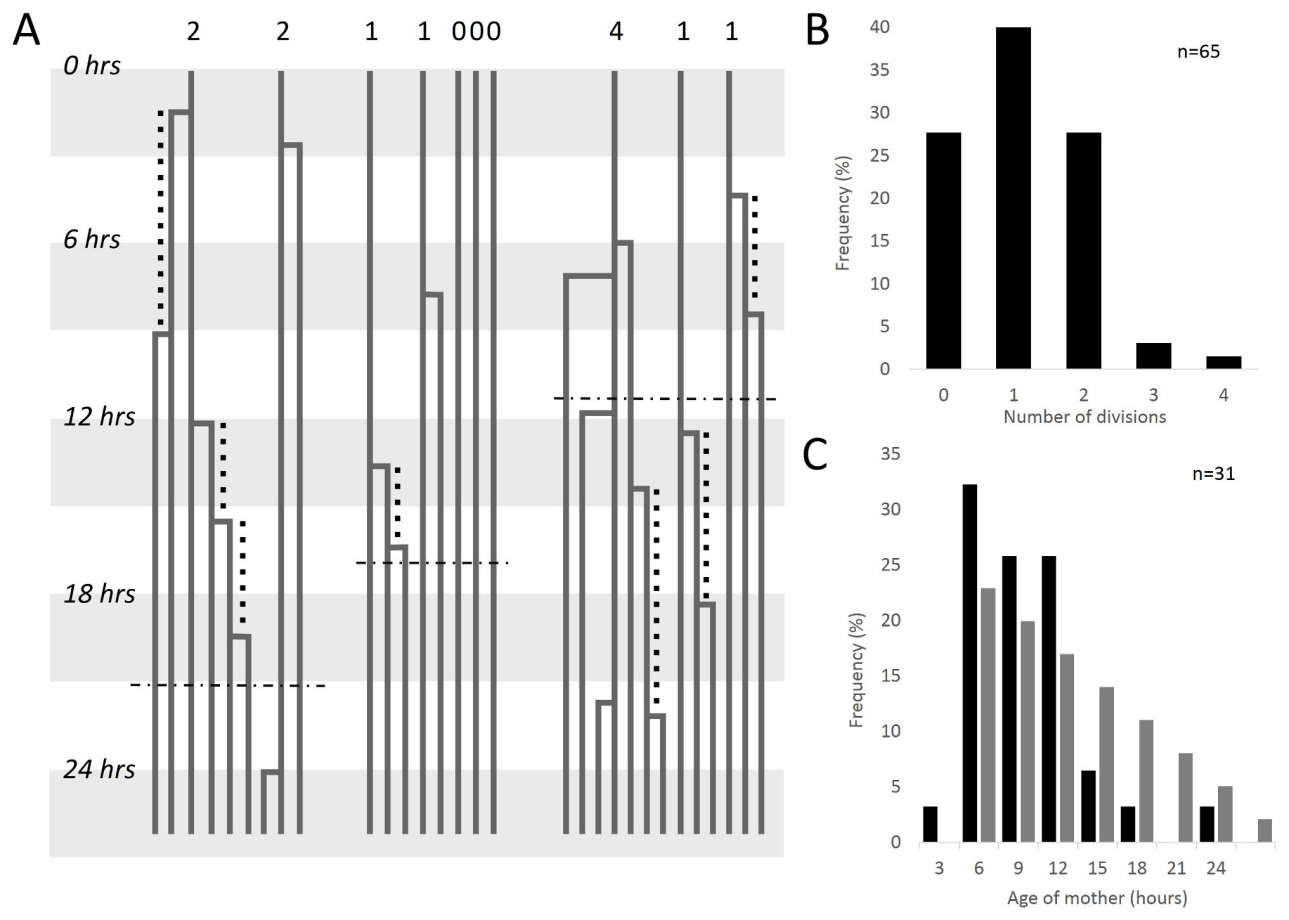
Mapping of carboxysome lineages reveals that new organelles undergo an initial refractory period before producing daughters of their own. (A) Example lineages of carboxysomes from three out of 25 total cells analyzed from a 522 frame movie taken at 3 minute intervals over approximately 26.1 hours. Each line represents a carboxysome tracked through time, with right-angle connectors joining daughters to mothers. Digits at the top of the panel indicate the number of times carboxysomes present at the beginning of the movie have colocalized birth events over the course of the analysis (26.1 hours), represented in the histogram in panel B. Vertical dotted lines indicate the measurable age of mothers when a daughter appears, represented in the histogram in panel C. Horizontal dotted lines indicate the time of cell division. (B) Histogram of the number of births colocalized to original carboxysome in the entire dataset (n = 65). (C) Histogram of measurable ages of mothers tabulated over the entire dataset (n = 31).

The majority of new carboxysomes are generated at the site of preexisting carboxsyomes. Early after induction of RbcL-GFP, many unlabeled carboxysomes are still present in the cell, limiting our ability to determine whether all new carboxysomes colocalize with preexisting ones or arise at unrelated locations in the cell. To address this, we induced RbcL-GFP for 24 hours to label several carboxysomes in each cell, and then used particle tracking to generate lineage maps of carboxysomes (Figure 2). At the initiation of imaging, 65 “original” carboxysomes were present, and over the course of 26.1 hours, 106 new carboxysomes were formed. Of these, only two could not be assigned to visible mothers.

Furthermore, carboxysome birth events are spatially ordered, preferentially occurring at the quarter positions along the long axis of the cell (Figure 1J). These data are consistent with previous findings that cells have a mean of 3.7 carboxysomes positioned equally along their length by ParA (Savage, 2010). After birth, however, the new daughter carboxysome frequently localizes near the cell pole (Figure 1A and 1D). Quantification of RbcL-GFP intensity reveals that birth events are highly asymmetric, with an average daughter–mother intensity ratio of ~1:4 (n=141, Figure 1K). We also observe that some birth events are correlated with rapid motions of either mother or daughter or both (Video S1). Indeed, automated tracking of carboxysome velocities suggests that carboxysome velocity is variable, with the mean maximum speed of a carboxysome being over 60nm per minute (Figure S2C). This may be related to the approximately 100nm per minute movement of ParA, assuming a 3µm cell [16]. Analysis of individual tracks reveals that mean carboxysome velocity is higher in the first several hours after birth (Figure S2F).

**Figure 3:**
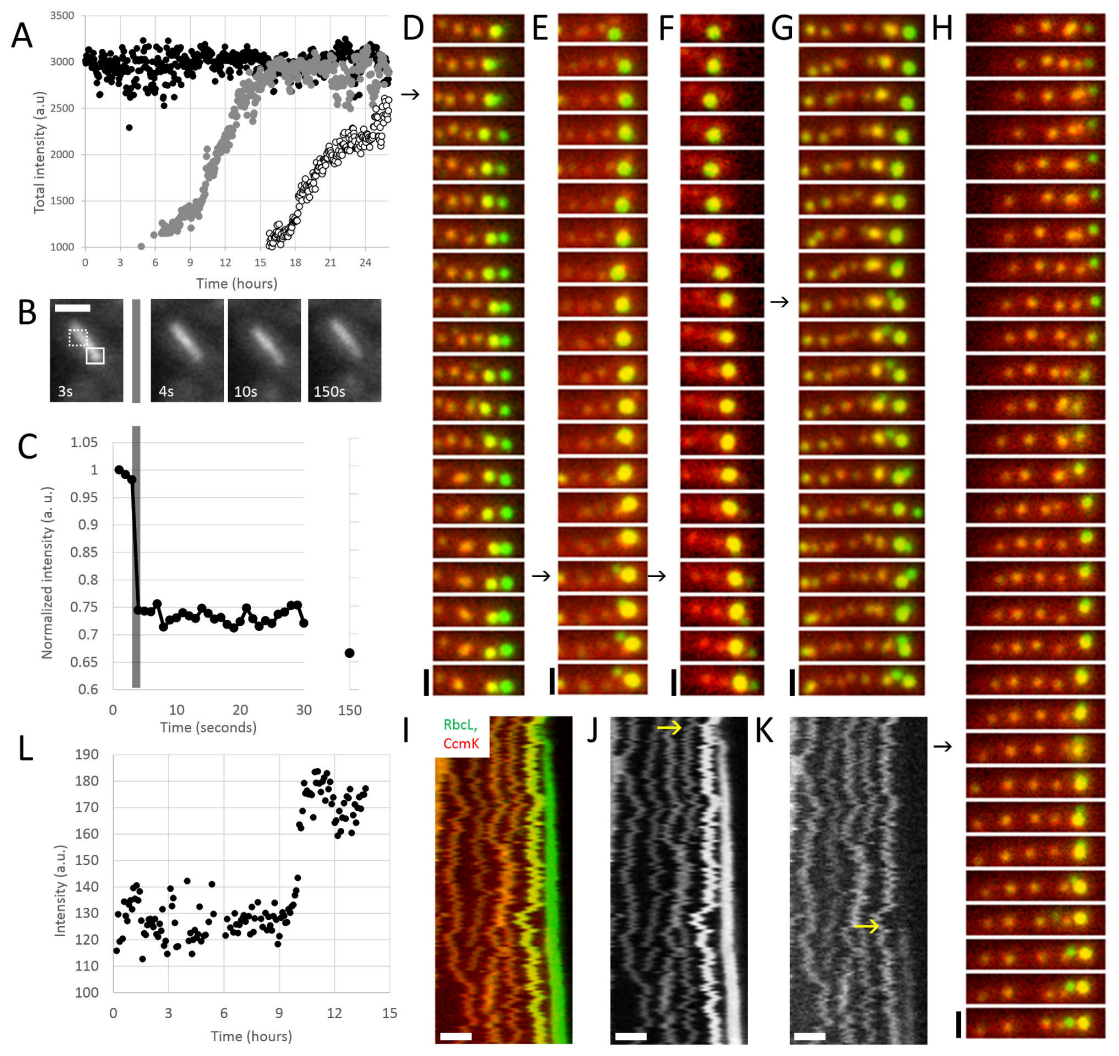
RuBisCO slowly forms a structured assembly prior to rapid colocalization of shell protein. (A) RuBisCO assembly, as measured by fluorescence intensity, follows sigmoidal kinetics. Each trace represents a new carboxysome. Cell is same as that depicted in Figure 1I. Imaging interval: 3 minutes. (B) Fluorescence recovery after photobleaching of a segment of a bar carboxysome. Solid box shows bleached area. Unbleached area (dashed box) was used for photobleaching correction. Cells were imaged at regular intervals after bleaching to assay for recovery. Scale bar: 1µm. (C) Quantification of FRAP in (B). Grey bar indicates bleaching event, when fluorescence sharply decreases. No recovery was seen after 150 seconds. (D–H) Time lapse of RbcL-mOrange (green) and CcmK4-GFP (red). Arrows indicate birth events of carboxysomes. Newly born RuBisCO initially buds off without shell protein. Shell protein colocalizes to RbcL-GFP foci hours after birth. In some cases (G and H), shell protein assembly is correlated with the formation of a new RuBisCO focus. Scale bar: 1µm. Time interval: 25 minutes. (I–K) Kymograph of RbcL-mOrange (J) and ccmK4-GFP (K) assembly. Shell protein assembly (yellow arrow in K) initiates well after RuBisCO birth event (yellow arrow in J). Scale bar: 1µm. Time interval: 5 minutes. (L) Individual trace of the fluorescence intensity of a CcmK4 focus in the process of formation. Time interval: 5 minutes.

**Figure 4:**
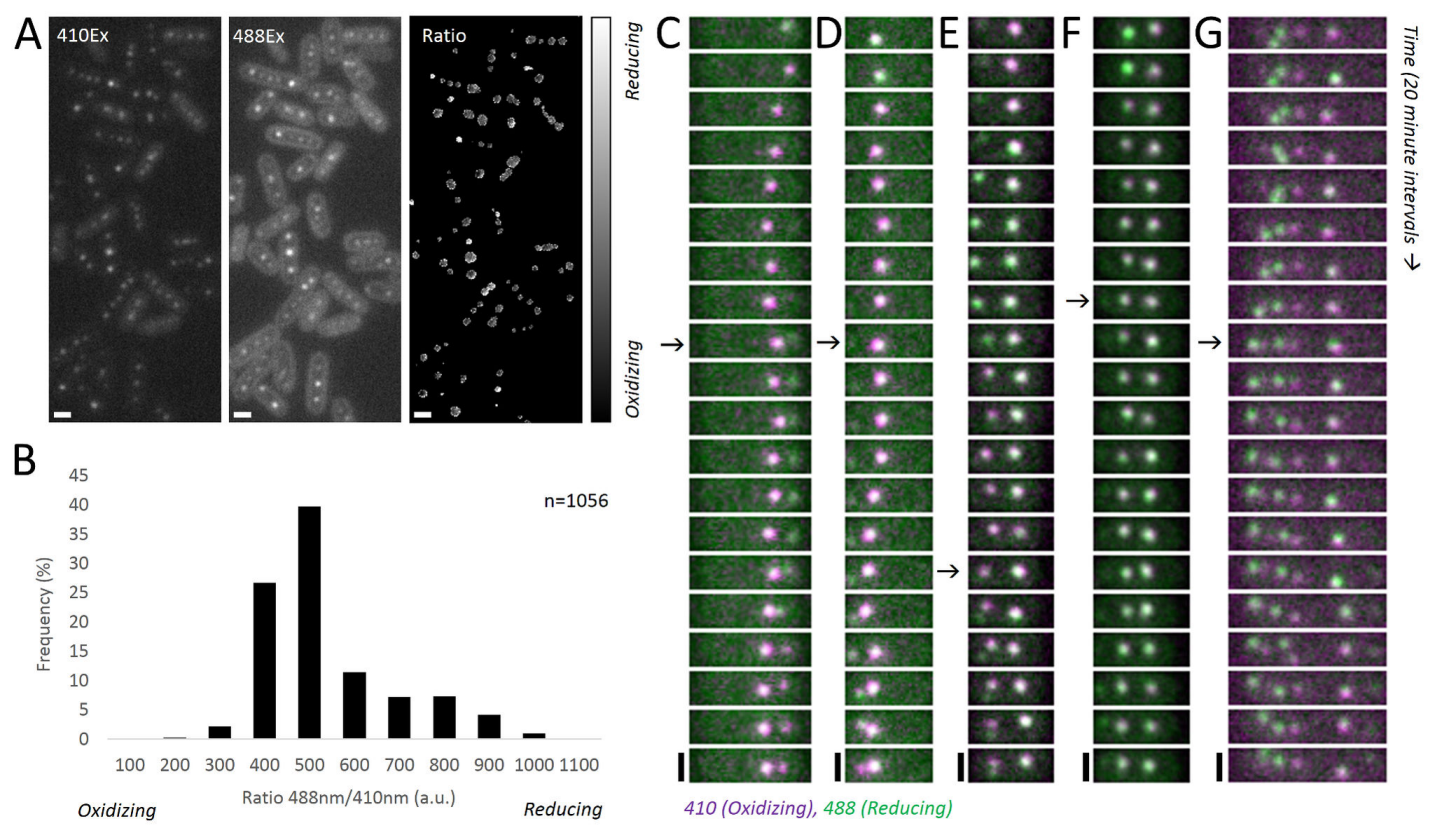
The carboxysome oxidizes over the course of its maturation. (A) RbcL-roGFP1 excited with 410nm (left) and 488nm (middle) produces ratiometric (488nm/410nm) differences in emission (right). Scale bar: 1µm. (B) A histogram of this ratio measured at each carboxysome focus reveals an asymmetric distribution biased toward a relatively oxidized state. (C–F) Montages of RbcL-roGFP1 show transitions from predominantly 488nm excitation (green) to 410nm excitation (magenta) over the maturation period of carboxysomes. Carboxysomes establish an oxidizing state before the appearance of a new carboxysome, rarely reopening to the cytosol after an initial closure (G). Arrows indicate birth events. Scale bar: 1µm. Interval: 20 minutes.

Elongated bar carboxysomes divide in two. These carboxysomes are well-documented by electron microscopy and are found in normal *Synechococcus* cells [13,17], though higher frequencies (up to 20% of all carboxysomes) are associated with environmental carbon limitation or mutations that compromise carbon fixation [14,18]. Their elongated morphology (1-3µm in length) provides a means to study the spatial organization of carboxysomes above the resolution limit of light microscopy. In our RbcL-GFP strain 0.5% of labeled carboxysomes are bars after induction with 25µM IPTG (n=191 total), and 1% are bars after 50µM IPTG (n=395 total). These carboxysomes colocalize with shell protein, though their redox state suggests that they are immature (Figure S3, compare to Figures 3 and 4). Bar carboxysomes begin as puncta that elongate and subsequently collapse or split into two carboxysomes (Figure 1E-F and Video S1).

### Maternal age influences the frequency of carboxysome births

By further analyzing the lineage maps, we found that new carboxysomes are more likely to be born near recently formed carboxysomes than near older ones. For each birth event, we measured the age of the mother carboxysome if it was born during the course of our observations (dotted lines in Figure 2A and histogram in Figure 2C). Strikingly, after a carboxysome is born, there is a marked refractory period until a new colocalizing daughter appears. This is characterized by a lack of births in the first three hours of its lifetime and suggests the structure must mature before another birth event occurs. Immediately following this refractory period, there is a burst of birth events. However, our imaging interval favors the observation of early birth events over late ones. To determine whether this apparent burst is significant, we compared our data to a model where birth probability is constant regardless of carboxysome age, adjusting for the limitations of our imaging interval. Our observed distribution of birth ages (black bars, Figure 2C) is significantly different from the theoretical distribution predicted by the age-independent model (grey bars, Figure 2C) (Kolmogorov-Smirnov test, h=1, p-value = 0.0416, k=0.2438). In comparison, our data show that birth rates are enriched in the first 12 hours of the lifetime of the mother. We measured the birth rate in the 9 hours following this three hour refractory period at 0.42 per carboxysome (n=57 carboxysomes), versus 0.28 per carboxysome over 9 hours for those at least 12 hours old (n=65).

While young carboxysomes have colocalized birth events more frequently during the burst period, mature carboxysomes have daughters randomly. By tabulating the number of times that carboxysomes visible at the start of the time lapse colocalized with new birth events, we found that this distribution of events is nearly Poissonian, with a mean of 1.1 and a variance of 0.8 (Figure 2B). This indicates that births near mature mothers is a random process. Furthermore, the probability that a preexisting carboxysome has daughters in the second half of the movie is not influenced by whether it did (0.36) or did not (0.37) in the first half of the movie, suggesting that births near mature carboxysomes are independent events. Furthermore, timing of carboxysome birth events is not correlated to cell divisions (horizontal dotted lines in Figure 2A). The distribution of birth event timing as a fraction of the cell cycle is not significantly different from a random uniform distribution across the cell cycle (Kolmogorov-Smirnov test, h=0, p=0.3222, kstat=0.13).

### Carboxysome biogenesis begins with a sigmoidal assembly of RuBisCO

To understand the nature of the maturation process, we followed the assembly of RuBisCO and coat protein (CcmK4). We first examined the kinetics of RuBisCO assembly by measuring the intensity of RbcL-GFP foci over time. This indicated that RuBisCO assembles over the course of many hours in distinct phases that display sigmoidal kinetics (Figure 3A). While individual carboxysomes assemble at different rates, we observed three regimes: a lag phase, followed by rapid assembly, and finally a plateau phase - assembly kinetics reminiscent of nucleation condensation polymers.

Once assembled, RuBisCO does not freely diffuse inside carboxysome foci. To probe the nature of assembled RuBisCO-GFP, we monitored fluorescence recovery after photobleaching (FRAP) of bar carboxysomes (Figure 3B-C and Figure S4). Bar carboxysomes are sufficiently large such that only a segment of the bar was bleached (Figure 3B solid box) while the rest of the bar remained fluorescent (Figure 3B dashed box). No recovery of fluorescence was seen up to 150 seconds after bleaching (Figure 3C). RuBisCO hexadecamers are roughly 500kDa in size, and freely diffusing protein complexes of similar molecular weight have been reported to recover in *in vivo* FRAP experiments in less than two seconds [19]. This discrepancy indicates that assembled RuBisCO does not freely exchange with monomers in the cytoplasm or in the rest of the carboxysome; rather, assembled cargo is static on the timescale of minutes.

### Shell proteins rapidly colocalize with RuBisCO late in the assembly process

To determine the relative kinetics of RuBisCO and shell assembly, we performed time-lapse microscopy of cells expressing inducible RuBisCO fused to mOrange and the shell protein CcmK4 fused to GFP under a constitutive promoter. At the beginning of the observation interval, recently born carboxysomes show strong signal in the RuBisCO channel, while old carboxysomes show weak, background levels of signal. New carboxysomes begin assembly with little to no detectable shell protein (Figure 3D–H and Video S2). Instead, shell protein associates with nascent carboxysomes at a later point: the mean time between the first appearance of RuBisCO and detectable shell protein was 4.7 hours (+/-2.2 hours, n=54). Kymographs of the formation process are shown in Figure 3I–K, where the shell protein suddenly colocalizes 8 hours after the birth event of the RbcL-GFP focus. The assembly of shell protein completes rapidly in contrast to the many hours required for RuBisCO assembly (Figure 3A); shell intensity reaches steady state in less than two hours (Figure 3L and Figure S5). Interestingly, in a fraction of cases, we observed the apparent birth of small daughter focus from a carboxysome 3.1 hours (+/- 1.1 hours, n=9) after detectable shell colocalized with the mother (Figure 3D–H). The timing of these events correlates with the burst of births following a maternal refractory period in lineage maps (Figure 2C).

### The carboxysome establishes a unique microenvironment late in the assembly process

The enzymatic activity of carbonic anhydrase relies on an environment more oxidative than the bacterial cytosol [20]. This predicts that the carboxysome must maintain an internal oxidizing state. To monitor changes in the redox state of the carboxysome over time, we tagged RuBisCO with the redox-sensitive roGFP1 [21]. The excitation spectrum of this protein shifts from one dominated by a maxima at ~488nm under reducing conditions to one dominated by a maxima at ~410nm under oxidizing conditions. By measuring the ratio of these two channels, we observed that carboxysomes display varying redox states within the same cell (Figure 4A). Over the entire population, we find that carboxysomes are distributed across a range of redox states (Figure 4B) but that the distribution is skewed toward oxidizing states (median=440, mean=487).

The late assembly of shell proteins on nascent carboxysomes predicts that maturing foci of RuBisCO share the reducing cytosolic environment. Indeed, monitoring changes in redox state revealed that newly formed RuBisCO foci are relatively reduced, regardless of whether imaging is started 6 (Figure 4C-D) or 24 (Figure 4E–G) hours after induction. As the carboxysome matures, RuBisCO-roGFP1 oxidizes, indicating the establishment of a distinct microenvironment (Video S3). Though the low signal from roGFP1 prohibits the time resolution required to measure the carboxysome lineage, the most recently synthesized carboxysome typically oxidizes before a new one appears (Figure 4C–F). Rarely, a carboxysome is apparently born from a mother rapidly switching between oxidized and reduced states (Figure 4G). Taken together, our data show that new carboxysomes are born concomitant with shell closure and establishment of the oxidizing microenvironment in the previously synthesized carboxysome.

### Carboxysomes persist over the cell cycle and their cargoes can be redistributed to daughter carboxysomes

**Figure 5:**
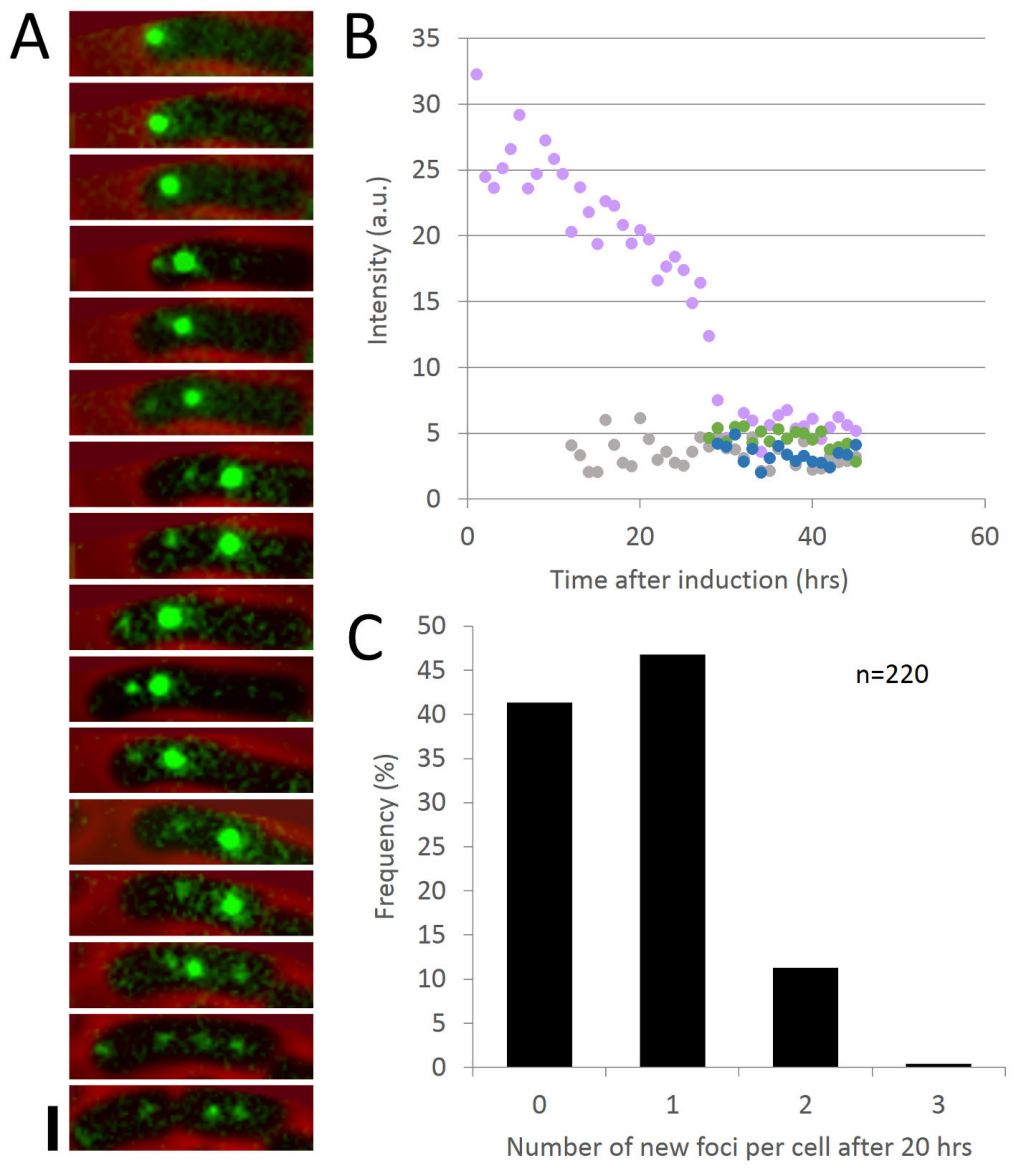
A solvent-accessible dye pulse labels foci that subsequently divide, but do not dissipate. (A) Montage showing one labeled RuBisCO focus partitioning into two or more daughter carboxysomes and persisting over the time interval of the experiment. Red: phase contrast. Green: RbcL-SNAP. Scale bar: 1µm. Time interval: 2 hours. (B) Intensity of the mother carboxysome (pink trace) sometimes decreases when new daughters (green and blue traces) are born. In other cases, the decrease is not detectable (grey trace). (C) Distribution of the number of new carboxysome foci formed per cell over the course of an experiment (20 hours). RuBisCO foci either persisted or divided over 20 hours (n = 220). All original foci were detectable at the end of the experiment.

We used the BG pulse-chase experiments (Figure 1A-B) to track the lifetime and fate of RuBisCO assemblies over days. We observed that labeled RuBisCO from one initial focus can partition into two or more daughter carboxysomes, and that it persists over the time interval of the experiment (45hrs) (Figure 5A). In some cases, the intensity of the mother carboxysome could be seen to decrease with the birth of a new focus (Figure 5B), indicating repartitioning of RuBisCO to new carboxysomes from old ones. In other cases, no decrease in mother intensity was detectable with the appearance of other BG foci. This is perhaps due to either splitting events with signal changes beneath our detection limit or the assembly of residual labeled cytosolic RuBisCO (Figure 5B, grey trace). During the period of this pulse-chase experiment, the number of labeled foci per cell was either invariant or increased (Figure 5C). Interestingly, disappearance of foci was never observed, suggesting that carboxysomes are not degraded, but rather passed down through generations.

## Discussion

We show that the *in vivo* biogenesis of carboxysomes occurs by preferential assembly on preexisting RuBisCO structures that later separate from mother carboxysomes. These stable, cytosol-accessible nuclei grow over a period of hours until shell proteins abruptly enclose the carboxysome, establishing a microenvironment distinct from the cytosol. This maturation can be coincident with the release of a new colocalizing daughter seed of RuBisCO.

Carboxysomes are the major carbon-fixing centers of the photosynthetic cyanobacterium *S* *. elongatus* ; thus, maintaining an appropriate number of organelles is vital to the cell [16]. The assembly of these compartments is regulated in two ways: 1) by formation of one carboxysome at a time, and 2) by regulation of their geometry. Our data suggest that both of these constraints arise from the assembly properties of the components.

### Cargo assembly is the primary organizer of carboxysome biogenesis

Carboxysome biogenesis is tuned to produce one structure at a time (Figure 1B). This may be an energetically efficient strategy, as focused assembly minimizes the net time carboxysomes spend in an incomplete state, during which they cannot deliver energetic benefits to the cell. In order to achieve focused assembly, there must be a kinetic barrier to spontaneous nucleation of cargo so that growth occurs only on preformed seeds.

Several lines of evidence support a nucleation-limited assembly mechanism of RuBisCO. First, the one-at-a-time assembly process suggests that templated assembly is favored over *de novo* nucleation. Second, the sigmoidal kinetics of RuBisCO assembly are reminiscent of nucleation-limited polymers. Third, the elongation of some RuBisCO seeds into bar carboxysomes suggests that this assembly is an extensible process, capable of producing structures far larger than mature icosahedral carboxysomes. Fourth, FRAP of bar carboxysomes demonstrates that assembled RuBisCO does not freely diffuse, again reminiscent of a polymer lattice with stabilizing interactions between neighboring subunits.

Previous work also supports this idea. Contents from purified carboxysomes can self-assemble in a concentration-dependent manner *in vitro* [14]. Furthermore, RuBisCO inside carboxysomes is organized into a lattice [13,22,23] implying that multiple self-associating interactions direct cargo to fill the interior layers of the carboxysome.

### Shell assembly specifies organelle size and limits further addition

Polymerized cargo appears to be stable and capable of extending far beyond the geometry of a mature icosahedral carboxysome, as suggested by the existence of bar carboxysomes. However, most mature carboxysomes are homogeneous in size. We propose that the rapid enclosure by the shell protein not only limits further cargo assembly by isolating the assembled RuBisCO from the cytosolic pool of subunits, but also sets the size of the carboxysome. Our data suggest two mechanisms for the size determination of RuBisCO assemblies: 1) size-selective enclosure, and 2) bisection of excess cargo.

We observe the assembly of shell protein only late in the biogenesis process (Figure 3I–K), presumably when the RuBisCO lattice reaches a given size. The topology of the growing RuBisCO seed thus would present a multivalent binding surface, with curvature depending on the size of the overall assembly. It is known that shell protein also self-associates into structures of a given radius [15]. Therefore, we speculate that when the curvature of the RuBisCO assembly matches that of the shell, RuBisCO-shell interactions organize shell-shell interactions, facilitating the assembly process. In other words, the intrinsic structure of the shell may act as a topological sensor that regulates timing of RuBisCO enclosure, ensuring that nascent carboxysomes reach a minimum size before encapsulation.

**Figure 6:**
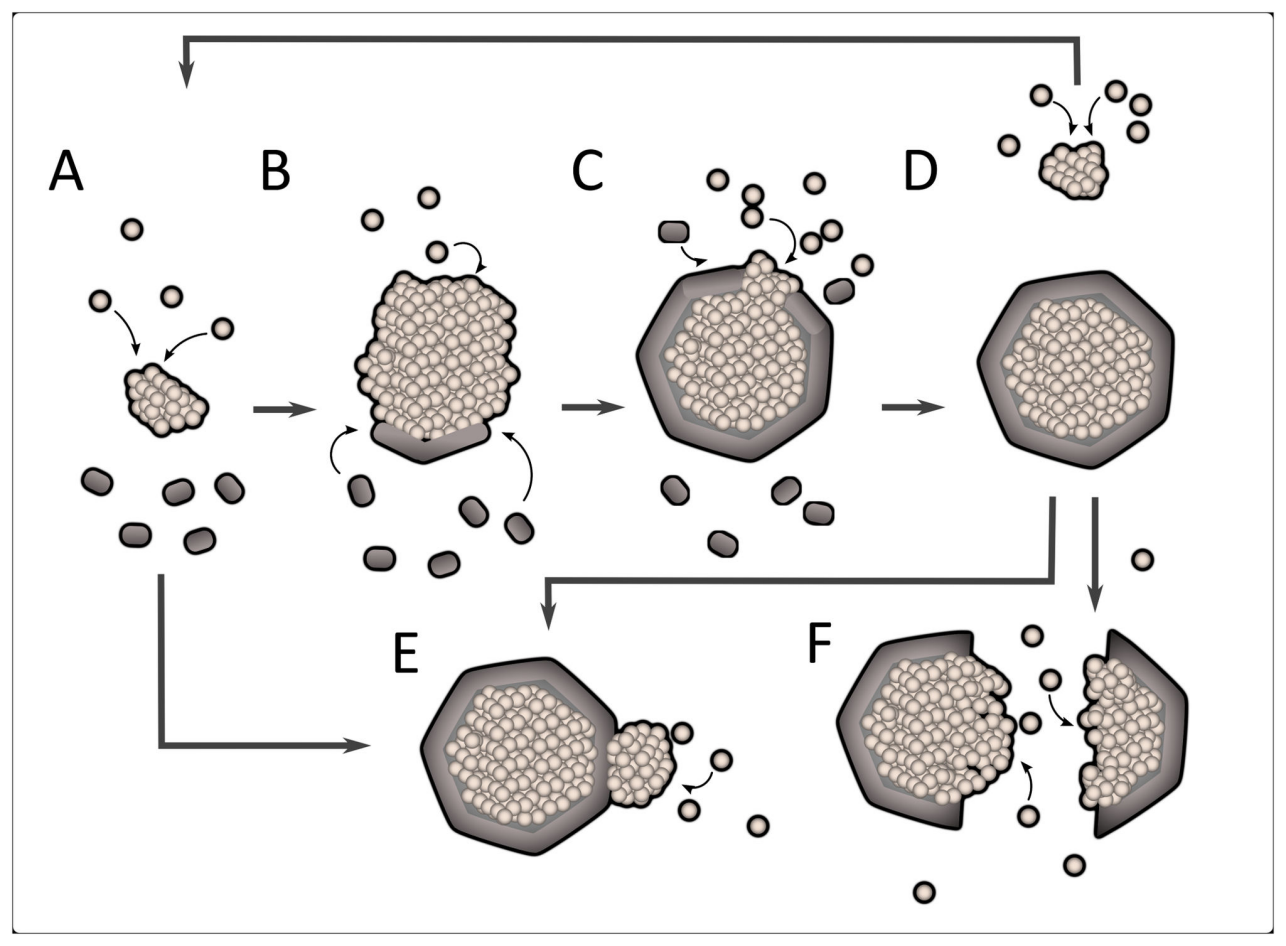
Model of carboxysome assembly. (A) RuBisCO seeds assemble from protomers over time. (B) Late in the assembly process, shell proteins rapidly assemble around RuBisCO. (C) Shell closure completes the carboxysome to establish an oxidizing environment, sealing RuBisCO from the cytosol. (D) A new RuBisCO nucleus forms after completion of the previous carboxysome. Colocalization may be driven by bisection of excess cargo by shell closure, or (E) by affinity of RuBisCO assemblies initiated elsewhere to the outside of the shell. (F) Rupture of a complete carboxysome would expose old RuBisCO cargo to template new assembly.

We also speculate that the polymerization of the shell can bisect a RuBisCO assembly to generate a mature carboxysome and a new RuBisCo seed (Figure 6C and D). This shell-mediated pinching hypothesis presents a parsimonious explanation for the increased birth rates from young mothers (Figure 2) and the coincidence of new seed formation with both shell association and the establishment of a distinct microenvironment in the mother (Figures 3 and 4). Our data also support non-pinching mechanisms of templated carboxysome replication. For example, our pulse-chase data indicates that mature carboxysomes can fracture, as we observe repartitioning of RuBisCO to two daughter carboxysomes (Figure 6F).

While our data support some forms of replicative biogenesis, our methods cannot discriminate between *de novo* and templated nucleation events. It is plausible that RuBisCO seeds assembled *de novo* may be brought into close proximity with preexisting carboxysomes by other mechanisms. The carboxysome itself may be sufficient to capture independent seeds: crystal packing evidence from other studies suggests that shell proteins may contact one another face-to-face or assemble into antiparallel strips [11,24]. This may expose cargo-interacting surfaces to the outside of the carboxysome, creating affinity for cargo on the exterior as well as the interior carboxysome surface (Figure 6E).

In summary, all proposed mechanisms rely on the self-association of RuBisCO as the primary organizer and driving force of carboxysome biogenesis, with shell protein defining organelle geometry.

### Broader implications

In addition to being crucial for global carbon fixation, the carboxysome has been proposed as a potential protein nano-factory capable of compartmentalizing heterologous reactions for metabolic engineering purposes [25]. An N-terminal peptide has been identified for the targeting of cargoes to 1,2-propanedeiol utilization microcompartments [26], but such a mechanism in carboxysomes has been elusive. Our studies of an assembly process dependent on self-association of cargo and the establishment of a unique internal microenvironment will inform the design of any future systems. 

## Materials and Methods

### Bacterial strains and growth conditions

A table of all relevant strains and plasmids is presented in Table 1. All chemicals were obtained from Sigma-Aldrich unless otherwise noted (St. Louis, MO). The wild-type *Synechococcus* *elongatus* PCC 7942 strain was acquired from the American Type Culture Collection (ATCC, Manassas, VA). *S* *. elongatus* cells were grown in solid BG11 medium with an illumination of 2000 lux at 30°C [27]. *S* *. elongatus* were transformed following standard protocols by washing with 10mM sodium chloride followed by incubation overnight in the dark with 100 ng of plasmid DNA and subsequently plating on selective media [28]. Antibiotics were used at the following concentrations: kanamycin 10 µg/ml, spectinomycin 50 µg/ml, and chloramphenicol 10µg/ml. 25µM isopropyl β-D-1-thiogalactopyranoside (IPTG) induction was used for RbcL-GFP or RbcL-mOrange. 50µM IPTG was used to induce formation of bar carboxysomes and 1mM IPTG for RbcL-roGFP.

**Table 1:** Bacterial Strains and Plasmids.

**Strain or Plasmid**	**Relevant genotype**	**Resistance**	**Reference**
*E coli* *strains*			
DH5-α	Host strain for plasmid construction		
*S* *. elongatus* *strains*			
PCC 7942	Wild-type *Synechococcus* , ATCC organism 33912		(Allen 1968)
RuBisCO/shell protein two color	Papca::ccmk4::sfGFP inserted in neutral site 1; lacI and ptrc::rbcL::mOrange2 inserted in neutral site 2	Kan/Sp	This work
*Plasmids*			
pDFS724	rbcL::sfGFP cloned into Neutral Site 2 at XbaI and NotI sites	Kan	(Savage 2010)
pDFS594S	Papca::ccmk4::YFP cloned into Neutral Site 1 at SpeI and NotI sites	Sp	(Savage 2010)
pAHC003	rbcL::mOrange2 cloned into pDFS724 in place of rbcL::sfGFP	Kan	This work
pAHC134	Papca::ccmk4::sfGFP	Sp	This work
pAHC126	rbcL::SNAP cloned into pDFS724 in place of rbcL::sfGFP	Kan	This work
pAHC149	rbcL::roGFP1 cloned into pDFS724 in place of rbcL::sfGFP	Kan	This work

### Plasmid construction

Cloning was done using Gibson assembly unless otherwise noted. IPTG inducible GFP strain (pDFS724) was obtained as previously described [16]. This neutral site 2 (NS2) plasmid contains a region with lacI and a promoter from pTRC99a followed by RbcL-sfGFP. sfGFP in pDFS724 was replaced by mOrange2 to obtain pAHC003. The shell protein fusion pAHC134 was obtained by modifying pDFS594s [16], replacing YFP with sfGFP. Two color strains were obtained by double transformation of pAHC003 and pAHC134. RbcL-SNAP fusion plasmid pAHC126 was obtained by replacing sfGFP in pDFS724 with SNAP tag. RbcL- roGFP fusion plasmid pAHC149 was constructed using restriction cloning at NheI and NotI. sfGFP in pDFS724 was replaced with roGFP1 (University of Oregon Remington Laboratory).

### Image acquisition

Cells were plated onto BG11 + 2% agarose pads with IPTG as necessary and placed on a glass bottom dish (Part No. P35G-1.5-20-C, MatTek, Ashland, MA). The addition of 100µl of water around dish edges and a paraffin film seal permitted long-term imaging.

FRAP image acquisition was performed on a Nikon Ti inverted microscope (Nikon Instruments, Melville, NY) with a MicroPoint laser targeting system (Photonics Instruments, Saint Charles, IL) controlling a 100mW solid state 488nm laser for photobleaching. Imaging was performed using a 100x 1.4 numerical aperture objective, an EXFO XL-120 (Lumen Dynamics Group, Mississauga, Canada) fluorescence light source, and an ORCA-R2 charge coupled device camera (Hamamatsu Photonics, Hamamatsu, Japan).

As previously described [16], all other imaging was done using a Nikon TE-2000 microscope with a 100x 1.4 numerical aperture objective, a Lumencor LED fluorescence illuminator, and an ORCA-ER (Hamamatsu Photonics, Hamamatsu, Japan) charge coupled device camera. Acquisition was controlled using a custom MATLAB script controlling µManager [29] and a network AC power controller (IP Power 9258T) for photosynthetic lighting. Images were processed and analyzed with ImageJ.

### Image analysis

For carboxysome lineage mapping, we used uTracker [30] to identify and localize closely-spaced point spread functions. The coordinates of these particles were imported into TrackMate, a plugin for FIJI [31] for manual annotation of track splitting events. Plots of lineages were retraced into vector format for counting division events and measuring maternal age.

For FRAP quantitation, the photobleaching rate after background subtraction was approximated with a linear function, which was used to correct measurements of the bleached region. Intensity was normalized to the maximum (in frame 1).

For ratiometric imaging, we background subtracted both 410nm and 488nm images with a 50-pixel radius rolling ball. We then registered the images with translations measured from imaging fluorescent beads and divided the 488nm image by the 410nm after converting to 32 bit format for floating point operations. This images was then multiplied by a mask of carboxysomes we generated based on a thresholded, 10-pixel rolling ball radius background-subtracted 410nm image. The mean intensities of regions larger than 9 pixels^2^ were quantitated with the “analyze particles” features of FIJI.

### Pulse-chase SNAP dye labeling

RbcL-SNAP strains that were induced with 25µM IPTG for 12 to 24 hours were labeled with SNAP-Cell BG 505-Star (New England Biolabs, Ipswitch, MA) following manufacturer’s instructions. Briefly, 1mL of cells were spun down and resuspended in 100 µL BG11 with 25µM IPTG and 5µM dye substrate. Labeling was done for 30 minutes in light. Cells were washed 3 times with BG11 and resuspended in BG11 with 25µM IPTG for 30 minutes in light. Cells were washed once more with BG11 and then transferred to an agarose pad for imaging. Either time-lapse imaging at 1 hour intervals or two endpoints 1 day apart were taken. Analysis was performed by manually counting and measuring foci intensity in ImageJ.

### Western Blotting

Cells were lysed by sonication in 3% SDS lysis buffer, and proteins were separated on NuPAGE Novex 4-20% Tris-glycine gels (Life Technologies, Grand Island, NY). Transfer to a nitrocellulose membrane was performed using the iBlot Gel Transfer Device and iBlot Gel Transfer Stacks (Life technologies, Grand Island, NY). Subsequent blotting was done using the SNAP-ID Protein Detection System (EMD Millipore, Bellerica, MA) following manufacturer’s instructions. Polyclonal anti-RuBisCO antibody (Agrisera, Prod. ID AS03 037) was used at a final dilution of 1:5000 and an HRP-conjugated goat anti-mouse antibody (Abcam, ab97265) was used at a final dilution of 1:5000. Peroxidase conjugates were detected using SuperSignal West Dura Exteded Duration substrate (Thermo Scientific).

## Supporting Information

Figure S1 **Quantification of RbcL and RbcL-GFP levels by Western blot.** The inducible RbcL-GFP strain was grown in the presence or absence of 25µM IPTG at early log phase for 12 hours. Using a rabbit polyclonal anti-RuBisCO antibody, the intensities of bands above background were quantified; the RbcL-GFP band is 11% of the intensity of the endogenous RbcL band.
(JPG)

Figure S2 **Mean and maximum velocities of carboxysome motion.** 126 carboxysomes were tracked over a minimum of 85 frames, and the mean and maximum velocity of each track quantified. (A) The tracks overlaid on one frame of the movie. Different colors represent different tracks. (B) The mean of the mean carboxysome velocity is 11.5nm/minute. (C) The mean of the maximum carboxysome velocity is 60.6nm/minute. (D) Velocity is variable across each track, with a subset showing maximal velocity near the start of the track. (E) 25 tracks were selected at random from this set, and velocity was plotted against the frame number (ie, age) of each track. Interval of acquisition, 5 minute. (F) The mean velocity per frame number across these 25 tracks. As track length is variable, fewer data points contribute to the mean toward higher frame numbers.
(TIFF)

Figure S3 **Bar carboxysomes colocalize with shell but are not oxidized.** (A) Bar carboxysomes contain both RuBisCO and shell protein. Red, CcmK4-GFP. Green, RbcL-mOrange. Scale bar, 1µm. (B) Bar carboxysomes are relatively reduced compared to punctate carboxysomes. Still frame composite images of 488nm (reducing, green) and 408nm (oxidizing, purple) RbcL-roGFP1 as in Figure 4. Scale bar, 1µm.
(TIF)

Figure S4 **Additional bar carboxysome FRAP data.** Bleaching events are indicated by grey lines. Unbleached portions of the bar were used to correct for photobleaching.
(TIFF)

Figure S5 **Additional shell protein assembly data.** (A–C) Individual traces of the fluorescence intensity of CcmK4 foci. Each panel represents a different cell, and only shell foci in the process of assembling are represented. Time interval: 5 minutes.
(TIF)

Video S1 **Normal and bar carboxysomes are born from replicative events.** Division events occur 2 seconds after the appearance of a white asterisk ~2µm above the relevant carboxysome. Imaging was initiated ~1 hour after induction. Green: RbcL-GFP. Red: phase contrast. Scale bar, 2µm. Frame rate, 12 frames (5 minute)/second.
(AVI)

Video S2 **Shell protein is late to localize to RuBisCO assemblies.** Shell localization events occur 2 seconds after the appearance of a white asterisk ~1µm above the relevant carboxysome. The top-most highlighted carboxysome also nucleates a daughter at the 12 hour mark. Imaging was initiated ~3 hours after induction. Green: RbcL-mOrange. Red: CcmK4-GFP. Scale bar, 2µm. Frame rate, 7 frames (5 minute)/second.
(AVI)

Video S3 **Nascent, reduced carboxysomes oxidize as they mature.** Oxidation events occur 2 seconds after the appearance of a white asterisk ~1µm above the relevant carboxysome. Imaging was initiated ~24 hours after induction. Green: 488Ex RbcL-roGFP1. Magenta: 410Ex RbcL-roGFP1. Scale bar, 2µm. Frame rate, 7 frames (10 minute)/second.
(AVI)
