# Supplementary figures and images for: The Bacterial Carbon-Fixing Organelle Is Formed by Shell Envelopment of Preassembled Cargo
Source: PLoS One. 2013 Sep 4;8(9):e76127. doi: 10.1371/journal.pone.0076127 (PMC3762834; doi:10.1371/journal.pone.0076127)

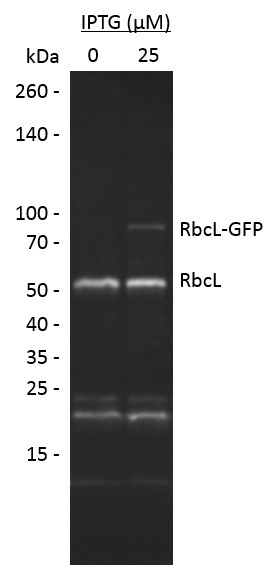

Supplement: Figure S1 — Quantification of RbcL and RbcL-GFP levels by Western blot. The inducible RbcL-GFP strain was grown in the presence or absence of 25µM IPTG at early log phase for 12 hours. Using a rabbit polyclonal anti-RuBisCO antibody, the intensities of bands above background were quantified; the RbcL-GFP band is 11% of the intensity of the endogenous RbcL band. (JPG) [file pone.0076127.s001.jpg]

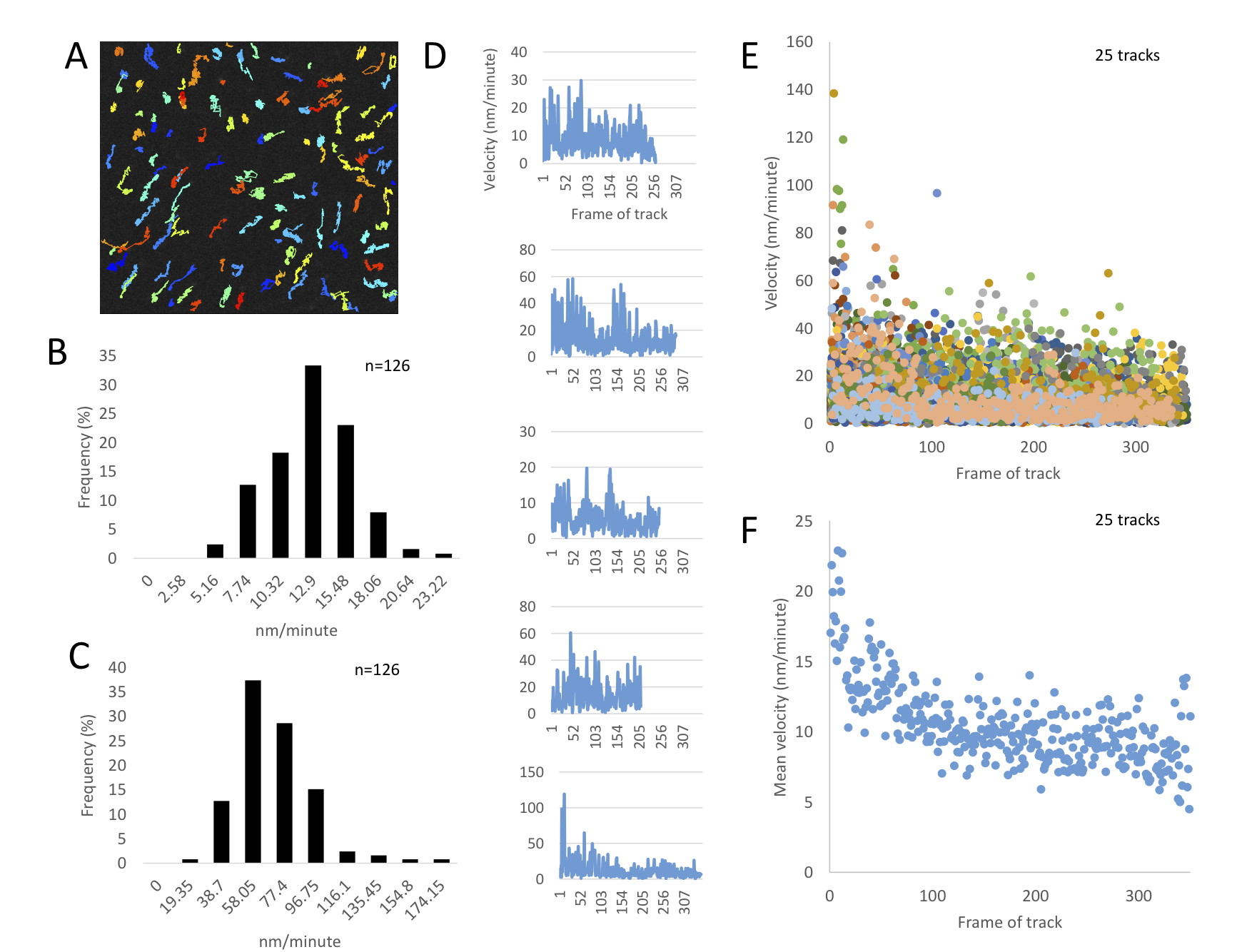

Supplement: Figure S2 — Mean and maximum velocities of carboxysome motion. 126 carboxysomes were tracked over a minimum of 85 frames, and the mean and maximum velocity of each track quantified. (A) The tracks overlaid on one frame of the movie. Different colors represent different tracks. (B) The mean of the mean carboxysome velocity is 11.5nm/minute. (C) The mean of the maximum carboxysome velocity is 60.6nm/minute. (D) Velocity is variable across each track, with a subset showing maximal velocity near the start of the track. (E) 25 tracks were selected at random from this set, and velocity was plotted against the frame number (ie, age) of each track. Interval of acquisition, 5 minute. (F) The mean velocity per frame number across these 25 tracks. As track length is variable, fewer data points contribute to the mean toward higher frame numbers. (TIFF) [file pone.0076127.s002.tiff]

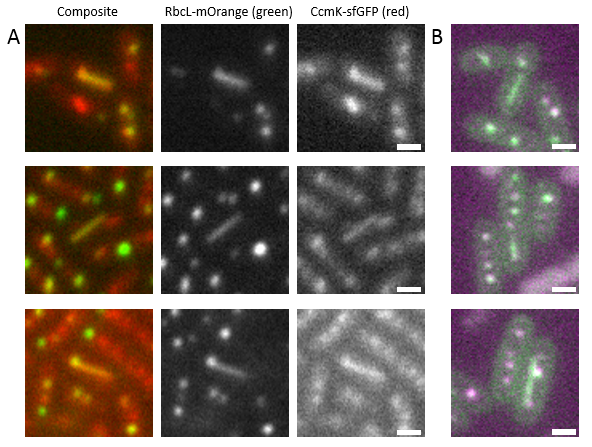

Supplement: Figure S3 — Bar carboxysomes colocalize with shell but are not oxidized. (A) Bar carboxysomes contain both RuBisCO and shell protein. Red, CcmK4-GFP. Green, RbcL-mOrange. Scale bar, 1µm. (B) Bar carboxysomes are relatively reduced compared to punctate carboxysomes. Still frame composite images of 488nm (reducing, green) and 408nm (oxidizing, purple) RbcL-roGFP1 as in Figure 4. Scale bar, 1µm. (TIF) [file pone.0076127.s003.tif]

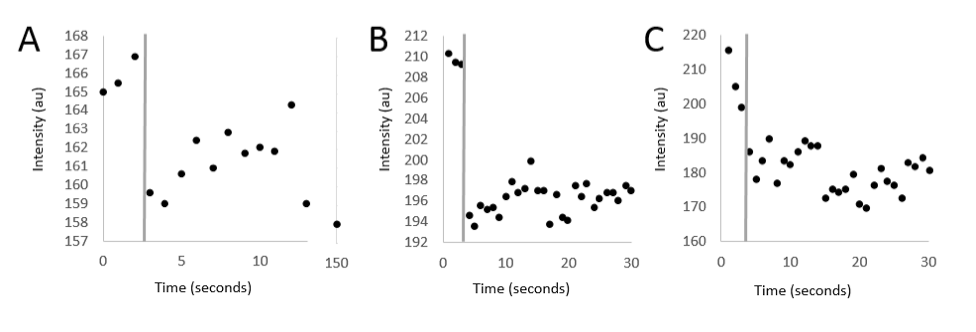

Supplement: Figure S4 — Additional bar carboxysome FRAP data. Bleaching events are indicated by grey lines. Unbleached portions of the bar were used to correct for photobleaching. (TIFF) [file pone.0076127.s004.tiff]

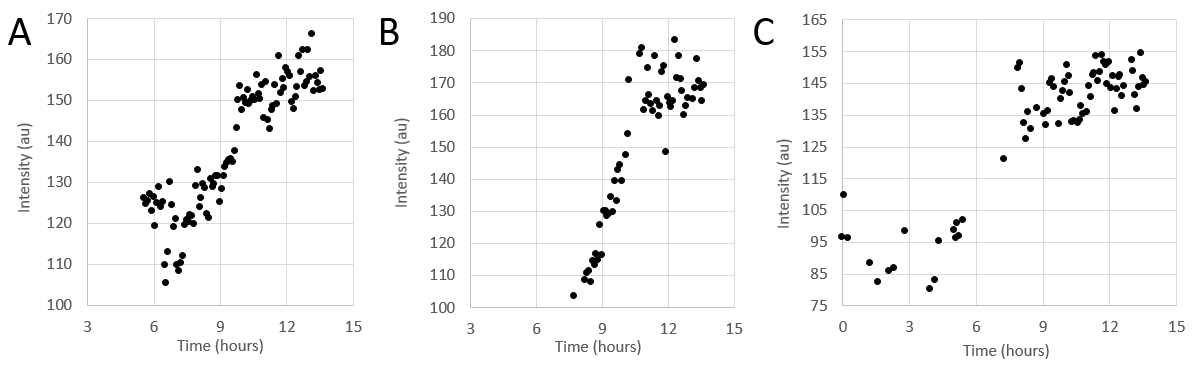

Supplement: Figure S5 — Additional shell protein assembly data. (A–C) Individual traces of the fluorescence intensity of CcmK4 foci. Each panel represents a different cell, and only shell foci in the process of assembling are represented. Time interval: 5 minutes. (TIF) [file pone.0076127.s005.tif]
